# Efficacy of a Methanolic Extract of* Adansonia digitata* Leaf in Alleviating Hyperglycemia, Hyperlipidemia, and Oxidative Stress of Diabetic Rats

**DOI:** 10.1155/2019/2835152

**Published:** 2019-03-07

**Authors:** Hossam Ebaid, Samir A. E. Bashandy, Ibrahim M. Alhazza, Iftekhar Hassan, Jameel Al-Tamimi

**Affiliations:** ^1^Department of Zoology, College of Science, King Saud University, Riyadh 11451, Saudi Arabia; ^2^Department of Pharmacology, Medical Division, National Research Centre, 33 EL Bohouth St., Dokki, Cairo, P.O. 12622, Egypt

## Abstract

Traditionally, in many countries, various parts of the* Adansonia digitata* (*A. digitata*) tree have been used in the treatment of many clinical ailments including diarrhea and dysentery. The phytochemical screening has indicated that the leaf extract of* A. digitata *contains flavonoids, saponins, mucilage, steroids, and alkaloids. Thus, this paper aims to evaluate the hyperglycaemic and hypolipidaemic effects of methanolic extract of* A. digitata* leaves (200 mg/kg and 400 mg/kg) in diabetic rats. The extract was administered orally for six weeks in the streptozotocin (STZ)-induced diabetic rats. The treatment with the extract caused a significant reduction in the blood glucose, glycosylated hemoglobin, cholesterol, triglycerides, low-density lipoprotein (LDL), interleukin 6 (IL-6), tumor necrosis factor-alpha (TNF-*α*), and malondialdehyde (MDA) levels by 46.7%, 46.15%, 48.91%, 43%, 60%, 66%, 45.45%, and 30.4%, respectively, as compared to the diabetic group after the sixth week of treatment. The leaf extract also mitigated the decline of high-density lipoprotein (HDL) level, RBCs count, hemoglobin level, packed cell volume (PCV %), and erythropoietin concentration in diabetic rats by 31%, 33.25%, 24.72%, 51.42%, and 220.68% with respect to the diabetic group. Also, the extract maintained the level of antioxidant enzymes, catalase (CAT) and superoxide dismutase (SOD), and reduced glutathione (GSH) in the diabetic rats. It also reduced the elevation in the white blood corpuscles (WBC) count in the STZ-induced diabetic rats. Our study, therefore, indicates that methanolic extract of* A. digitata *leaf exerts strong antidiabetic and hypolipidaemic properties in a dose-dependent manner by improving the hematological properties and redox parameters in the experimental diabetic rats.

## 1. Introduction

Diabetes mellitus is a metabolic disorder leading to severe organ damage and multiple organ failure as per the severity of the disease [1, 2]. The free radicals elicited during the pathogenesis of the disease also cause DNA damage besides the glycation of critical proteins, protein modification reactions, and lipid peroxidation [3]. The chronic version of this disease further exacerbates with persistent hyperglycemia, and increased oxidative stress causes various clinical complications compromising the quality of life in the affected patients [4, 5]. Despite the extensive research on its treatment for many decades, an effective treatment modality is still to be achieved.

Medicinal plants with a history of efficient use are likely to have a pharmaceutical outcome in diabetes.* Adansonia digitata* and its related species belong to the family of Malvaceae. The tree is of African origin known for its medicinal and nutritional value. It has excellent antioxidant and anti-inflammatory properties; various parts of the tree are used to treat different types of ailments [6, 7]. Recent phytochemical analysis of its leaves revealed that they contain a rich repertoire of reducing sugars, flavonoids, terpenoids, saponins, tannins, alkaloids, anthraquinones, steroids, resins, phenols, and cardiac-active glycosides [8]. Besides, the leaves have an abundant amount of mucilage, carbohydrate (60-70%), protein (13-15%), fiber (11%), fat (4-10%), and minerals including calcium, iron, potassium, magnesium, phosphorous, zinc, and manganese [9]. Previously, Talari et al. [10] had concluded that methanolic extract of various parts of the tree showed a strong free radical scavenging activity associated with their phytochemical constituents. This property raises the possibility that these methanolic extracts could be utilized in the preparation of antioxidant drugs for the treatment of a range of ailments [10].

In many countries, various parts of the tree have been traditionally used in the treatment of diarrhea and dysentery. Besides, different parts of the tree have been reported to have analgesic, immunostimulant, anti-inflammatory, insect repellent, and pesticidal properties [11, 12]. Intriguingly, the leaf extract has ten times more potent antioxidative capacity than vitamin C. Also, the leaf extract inhibits anti-inflammatory iNOS expression, which might be related to the elimination of peroxyl radicals as well as inhibition of signal transduction mediated by NF-*κ*B. Its leaf extract has significant antioxidant potential that might prevent malignancies related to inflammation [13]. The methanolic extract of the bark of its stem has previously been found to have a hypoglycemic activity in streptozotocin (STZ)-induced diabetes [14]. Further, separate studies indicated that the methanolic extract of the leaf and pulp of the plant also has a hypolipidaemic effect [15, 16] making its parts suitable for treatment of different ailments.

Moreover, in contemporary research, plant-derived drugs are considered less toxic and more compatible with biological systems because of their lesser side effects as compared to the synthetic ones. Hence, a great deal of scientific research is focussing on developing new drugs based on natural or nature-identical compounds of herbal origin for the treatment of complicated diseases like diabetes [17, 18]. In the present study, diabetes mellitus was induced in laboratory animals (rats) by STZ to mimic the pathophysiology of the disease in humans [19]. This investigation was designed to investigate the efficacy of the leaf extract on STZ-induced hyperglycemia, hyperlipidemia, and oxidative stress in the albino rats.

## 2. Material and Methods

### 2.1. Plant Material

The leaves of the tree (1500 g) were collected from Sudan, Kordofan, in February of 2016. The leaves were carefully identified by Mrs. Tersea Labib, a taxonomist at Orman Botanical Garden (Voucher number C 18), Giza, Egypt. The leaves were air-dried under the shadow with proper ventilation, and then they were ground finely in a mill.

### 2.2. Preparation of the Leaf Extract

The powdered leaves were extracted with 70% methanol at room temperature for five days with shaking and stirring. The extract was filtered and then concentrated to dryness in the rotary evaporator to obtain the crude extract. The final yield after all these processing was 135 gram (dried weight) of the extract.

### 2.3. Phytochemical Screening

The phytochemical screening of the extract was done according to AOAC methods [20]. The screening of the extract confirmed the presence of flavonoids, saponins, mucilage tannins, terpenoids, alkaloids, steroids, anthraquinones, and active glycoside (data not shown).

### 2.4. Chemicals

Streptozotocin (Sigma Chemical Co., USA) and glibenclamide (Hoechst Co., Egypt) were used in the present experiment. All other chemicals were of analytical grade.

### 2.5. Animals

Forty adult Wistar albino rats (male; 170-200 g) were purchased from the National Research Centre Laboratory (Dokki, Giza, Egypt). They were housed in the standard polypropylene cages under suitable environmental conditions with maintained light-dark cycles. They were adapted for one week at normal pellet diet and water* ad libitum*.

All the animals based experiments were carried out as per the recommendations of Guide for the Care and Use of Laboratory Animals of the National Institutes of Health (NIH publication No. 85–23, revised 1996). The sacrifice of the animals was performed under local anesthesia with ether. All efforts were made to minimize suffering during the experimentation on the animals.

### 2.6. Determination of Median Lethal Dose (LD_*50*_)

LD_50_ was calculated according to a method of Lork [21]. The LD_50_ of the extract in the rats was found as 4000 mg/kg by the oral route of administration.

### 2.7. Induction of Diabetes Mellitus

Diabetes mellitus was induced in overnight-fasted rats by a single intraperitoneal injection of freshly prepared STZ (50 mg/kg, dissolved in 0.1 M cold citrate buffer, pH 4.5) as described previously [22]. Rats were tested for successful induction of diabetes 2 days after STZ injection by determining fasting blood glucose levels by commercial glucometer (BIONIME GmbH, Switzerland). Rats with blood glucose levels >240 mg/dL were included in the study. The animals were further treated on the third day from STZ injection.

### 2.8. Experimental Design

Forty male rats were divided into five groups (*n* = 8) as follows:

 
*Group I:* normal control (negative control, CN)
 
*Group II:* positive control (diabetic untreated rats, DM)
 
*Group III:* diabetic rats treated with the leaf extract at 200 mg/kg body weight (1/20 of LD50)
 
*Group IV:* diabetic rats treated with the leaf extract at 400 mg/kg body weight (1/10 of LD50)
 
*Group V:* diabetic rats treated with glibenclamide at 5 mg/kg body weight [23]

The treatment with leaf extract or a reference drug was via oral route for 6 weeks.

### 2.9. Blood Samples

Blood samples from the treated animals were taken at 2 and 4 weeks. After six weeks of the treatment, two aliquots of blood samples were collected from a retroorbital vein in the heparinized tubes. One portion of the blood samples was used for blood cell count, and the plasma separated from the remaining portion was stored in the Eppendorf tubes at -30° for biochemical analysis. After removal of the plasma, the packed RBCs were bathed twice with an ice-cold isotonic physiological saline solution. Following that, a known volume of RBCs was lysed in cold phosphate buffer (pH 7.4). The hemolysate was separated by centrifuging at 3500 rpm for 10 min, at 2°C. The resulting hemolysate was used as samples for assessment of antioxidant enzymes, superoxide dismutase (SOD) and catalase (CAT).

### 2.10. Total Blood Count and Measurement of Glycosylated Hemoglobin

Blood cells and hemoglobin were counted by hematology analyzer (Scil Vet ABC, operations manual, USA) while glycosylated hemoglobin was estimated by a commercial kit (BioSystem SA, Barcelona, Spain) according to manufacturer's method.

### 2.11. Assessment of Oxidative Stress Parameters in the Plasma Samples

All the key oxidative stress parameters including malondialdehyde (MDA), glutathione (GSH), catalase (CAT), and superoxide dismutase (SOD) were determined using colorimetric kits (Bio-diagnostic, Egypt). SOD and CAT were expressed in unit per gram of hemoglobin (Hb).

### 2.12. Measurement of Immune System Parameters in the Plasma Samples

ELISA technique was used for the assessment of tumor necrosis alpha (TNF- *α*), interleukin 6 (IL-6) by the commercial kits (R&D Systems, USA). The measurement of the level of erythropoietin was also conducted by an Elisa kit (Cusabio Biotech CO, Hubei, China).

### 2.13. Lipid Profiling in the Plasma Samples

The lipid profiles including cholesterol, triglycerides, HDL, and LDL were measured colorimetrically by the kits (Salucea Company, Netherlands) in the plasma samples.

The parameters like body weight, blood glucose, and lipid profile were determined after treatment at the intervals of 2, 4, and 6 weeks while other parameters were evaluated at the end of the study time.

### 2.14. Statistical Analysis

All values have been expressed as mean ± S.E.M of eight independent samples from each group (n=8). Statistical analysis of data was performed using two-way ANOVA followed by the least significant difference (LSD) for comparison of various treatments using the 13.0 version of SPSS statistical analyzing software taking p values ≤ 0.05 to assess significant difference among the values. The values of the treatment groups were compared with the negative control (CN, group I), positive control (DM, group II) and the reference drug, glibenclamide. Few of the experiments were repeated to confirm the reproducibility of the results.

## 3. **Results**

### 3.1. Blood Glucose

**Figure 1:**
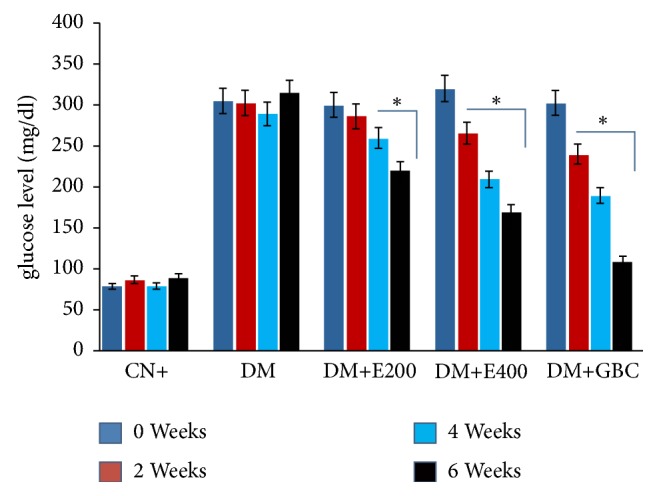
*Showing the glucose level of the indicated groups at 0, 2, 4, and 6 week of the treatment*. All the data has been expressed as mean ± SEM of eight independent samples from each group (*n* = 8) analyzed by SPSS 13.0 version. All the treatment groups were statistically significant from the normal control (CN or group I). *∗*: statistical difference from the positive control group, DM (group II). DM+E200: diabetic rats treated with 200mg/Kg of leaf extract (group III); DM+E400: diabetic rats treated with 400mg/Kg of leaf extract (group IV); DM+GBC: diabetic rats treated with glibenclamide (group V).

The fasting blood glucose levels of the diabetic rats (group II) were significantly higher than the normal control rats (group I) at all-time intervals taken in the study (Figure 1). The treatment of diabetic rats with leaf extract of* Adansonia digitata*, however, led to a significant decrease in glucose levels compared to group II. It is noteworthy that no significant difference in glucose level was observed between diabetic rats given 400 mg of leaf extract (group IV) and diabetic rats treated with the reference drug, glibenclamide (group V), till the second and fourth weeks. However, the glucose level in the rats treated with the reference drug was significantly less than that of the* Adansonia digitata *leaf extract group at the sixth week.

### 3.2. Body Weight

The body weight decreased significantly in group II as compared to group I (Table 1). Meanwhile, the body weight of groups V and IV did not change significantly as compared to group I at any interval of the treatment. The body weight of group III, however, was significantly reduced at the sixth week as compared to group I but appeared healthy at the second and fourth weeks. It is noteworthy that no significant difference was observed between groups IV and V.

### 3.3. Lipid Profile

The effect of* Adansonia digitata *leaf extract on the lipid profile of diabetic rats has been shown (Tables 2, 3, 4, and 5). The levels of cholesterol, triglycerides, and LDL were significantly higher in group II as compared to group I at all-time intervals of the experiment. On the other hand, HDL level of group II decreased significantly compared to group I after six weeks; however, it did not change significantly at the other time intervals. The treatment given to the diabetic rats with either a reference drug or* Adansonia digitata *leaf extracts led to a significant decrease in the level of cholesterol, triglycerides, and LDL compared with group II. With respect to cholesterol or HDL levels, no significant difference was observed between diabetic rats treated with a reference drug (group V) and diabetic rats who received 400 mg/Kg of leaf extract (group IV), although group V showed improved triglyceride and LDL levels than group IV.

### 3.4. Oxidative Stress Parameters

**Figure 2:**
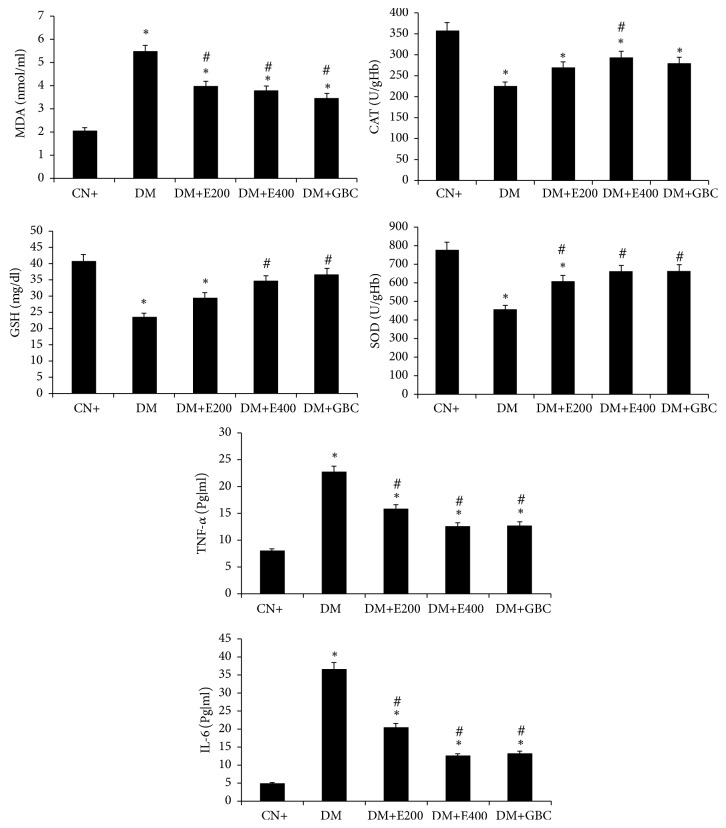
*Showing the level of MDA, GSH, CAT, and SOD and plasma inflammatory markers of the indicated groups after the treatment*. All the data has been expressed as mean ± SEM of eight independent samples from each group (*n* = 8) analyzed by SPSS 13.0 version. *∗*: statistical difference from the normal control (CN or group I). #: statistical difference from the positive control group (diabetic: DM or group II). DM+E200: diabetic rats treated with 200mg/Kg of leaf extract (group III); DM+E400: diabetic rats treated with 400mg/Kg of leaf extract; DM+GBC (group IV): diabetic rats treated with glibenclamide (group V).

Oral administration of leaf extract of* Adansonia digitata *to diabetic rats led to a significant decrease in malondialdehyde, TNF-*α*, and IL-6 in the groups III and IV as compared to group II (Figure 2). On the other hand, the leaf extract significantly enhanced GSH, CAT, and SOD levels in diabetic rats. No significant difference in oxidative stress parameters was observed between diabetic rats receiving 400 mg/kg leaf extract (group IV) as compared to those receiving the reference drug, glibenclamide (group V). However, the drug showed better results in the parameters including MDA, GSH, TNF-*α*, and IL-6 in group II than the leaf extract at a dose level 200 mg/Kg (group III), while no significant difference was observed in the values of CAT and SOD for the same dose of the extract (Figure 2).

### 3.5. Blood Cell Count, Glycosylated Hemoglobin, and Erythropoietin

The results of the present study indicate a significant decrease in RBCs count, Hb level, PCV (%), and erythropoietin concentration in group II (Table 6). On the other hand, a prominent increase in the WBC count and GHb was noticed in the same group. The treatment of diabetic rats with leaf extract of* Adansonia digitata *reduced the deleterious effect of STZ on blood cell count, GHb and EPO as evidenced by groups III and IV. No significant differences were observed between diabetic rats given 400 mg/Kg* Adansonia digitata *leaf extract and diabetic rats treated with glibenclamide regarding WBCs count, Hb level, PCV (%), and GHb; however, there was a remarkable difference in the RBCs count and EPO level. The reference drug, glibenclamide, caused more improvement in the previous parameters than the* Adansonia digitata *leaf extract at a dose level of 200 mg/kg (group III).

## 4. Discussion

The drugs derived from the medicinal plants are safe, less toxic, and lower-priced. Since ancient times,* Adansonia digitata *has been used extensively in various traditional medicines as well as a substitute for Western drugs [9]. It has been specifically reported that various parts of the tree possess an antidiabetic effect [14, 24]. In the present study, leaf extract of the tree showed a hypoglycemic effect which can be attributed to its active constituents, flavonoids, saponin, and mucilage. For example, Lee [25] showed that supplementation of flavonoids to STZ-induced diabetic rats led to an increase in the activity of insulin and glucokinase in the plasma samples concomitant with a decrease in the glucose-6-phosphatase activity. Besides, Bathena et al. [26] showed that some of the flavonoids present in the extract compete with glucose absorption through several absorption mechanisms. It might be one of the reasons leading to intestinal absorption reduction attributive in the hypoglycaemic effect. Furthermore, important ingredients of the extract, saponins [27] and mucilage [28], have been reported to have a strong hypoglycemic effect in the diabetic rats. Also, Rajkumar et al. [29] have suggested that the extract reduced weight gain in the diabetic rats which might be due to an elevated rate of catabolism leading to muscle loss. In this context, the present study confirms the earlier findings that oral administration of the leaf extract of* Adansonia digitata *improves the body weight in diabetic rats suggesting an improvement in their glycaemic control.

With the progression of diabetes mellitus, various types of disease-related complications including hypercholesterolemia and hypertriglyceridemia are quite common that further exacerbate the patient's health condition [30–32]. There is an uphill in the mobilization of free fatty acids during the progression of the disease leading to elevation of serum lipids abnormally in the diabetic patients. Consequently, the activity of hormone-sensitive lipase releases more free fatty acids into the serum [33], and an increase in fatty acids promotes conversion into phospholipids and cholesterol in the liver during the disease. These two substances along with triglycerides and various forms of lipoproteins may be discharged into the blood [34]. In our experiment, a significantly increased level of serum total cholesterol, triglycerides, and LDL and decreased HDL cholesterol were observed in the diabetic rats. However, the methanolic extract of* Adansonia digitata *leaf showed a hypolipidaemic effect in rats fed which can be attributed to its constituents- saponin and fiber [15]. Intriguingly, the methanolic extract of leaf of* Adansonia digitata *not only decreased the total cholesterol but also enhanced the HDL. Besides, it is also reported that the administration of* Adansonia digitata *extract reduces TG and LDL in the diabetic rats [35].

Furthermore, a great deal of literature entails that free radicals and oxidative stress are one of a causative factor in the etiology and progression of many diseases including cancer and diabetes [36]. Typically, free radicals are scavenged by antioxidant enzymes like SOD and catalase which protect the body from oxidative abuses [37, 38]. The decline in the activity of these redox markers can lead to an increase in superoxide anion, hydrogen peroxide, and hydroxyl radicals resulting in extensive lipid peroxidation in the diabetic patients [39]. In the present study, an increase in the oxidative stress in the diabetic rats could be attributed to the exhaustion of the antioxidant enzymes in attenuating the free radicals generated during the metabolism of STZ. Also, hyperglycemia leads to elevated oxidative stress in diabetic patients which involves extensive lipid peroxidation [40, 41]. The aldehyde groups of MDA formed during lipid peroxidation can act as an anchor between sugar and protein moieties which enhances the formation of glycated proteins [42]. It is possible that such glycation drive might also affect the antioxidant enzymes leading to the diseased condition. It is reported that leaf extract of* Adansonia digitata *is rich in flavonoids that are the phenolic compounds having hydroxyl groups in their structure enhancing their antioxidative activity [43]. These compounds might be attributive in improving the redox status in the diabetic rats treated with the leaf extract of* Adansonia digitata *[13] as evidenced in the present study. Besides, the methanolic extract of* Adansonia digitata *leaf has been reported to inhibit anti-inflammatory iNOS expression, which might eliminate peroxyl radicals in the free radicals mediated diseases [13]. This notion might be attributive in the reduction of the plasma inflammatory markers: TNF-*α* and IL-6 levels in the diabetic rats treated with the leaf extract in the current study. Regulation of oxidative stress concomitant with inflammatory markers in the diabetic condition could be of therapeutic relevance for the improvement or delay of the complications linked to chronic hyperglycemia.

In diabetic patients, many key biomolecules, such as hemoglobin and RBC membrane proteins, are modified by glycation [44]. This structural alteration may lead to impaired protein function and perhaps also contribute to the long-term complications of diabetes. Hence, glycosylated hemoglobin is considered a reliable marker of overall glycaemic control in the assessment of the individuals prone to diabetes [45]. Intriguingly, oral administration of the leaf extract decreased hyperglycemia and the level of glycosylated hemoglobin in the present investigation. Moreover, alterations of membrane proteins may also lead to RBC membrane hemolysis and anemia [46]. Herein, administration of the extract to diabetic rats was shown to alleviate the disease-induced decline in RBCs count and Hb level. The extract decreased not only serum glucose level but also the lipid peroxide resulting in a decreased susceptibility of RBCs to hemolysis. Moreover, the extract enhanced the erythropoietin level in diabetic rats increasing the RBCs count. Hence, the improvement in the RBC count, PCV, and Hb after administration of the methanolic extract of* Adansonia digitata *leaf may confirm the extract's positive effects on the hemopoietic system in diabetes-induced rats. Also, the results of the present study also indicated an increase of WBC count in diabetic rats. It is documented that the release of cytokines, such as TNF-*α* [47] and superoxide [48] may activate leukocytes. The diabetic mice in previous studies have shown moderate neutrophilic leucocytosis with prolonged circulation times of neutrophils and monocytes [49]. It is assumed that raised leukocyte count may also reflect low-grade inflammation. Hence, it is quite evident that the herb extract, in the present study, effectively ceases the glycation of Hb, lipids, and essential proteins as well as checks the inflammation significantly in the diabetes model rats.

The current investigation entails that the methanolic leaf extract of* Adansonia digitata* improved many parameters of oxidative stress, lipid profile, basic hematology, and immune system of the diabetic rats in a dose-dependent manner. The higher dose of the herb extract even showed results comparable to the reference antidiabetic drug, glibenclamide, in the rats. The herb extract demonstrated excellent hypoglycaemic and hypolipidaemic effects in the diabetic rats nullifying many of the signs of anemia and normalized diabetes-related hematological alterations. These effects are attributed to the components of the herb extract that can cease lipid peroxidation with the maintenance of cellular antioxidant enzymes and attenuation of proinflammatory cytokine production. Hence, the study pleads for the therapeutic potential of the herb extract in diabetic patients. The active ingredients of the herb can be role models for designing or developing novel drugs for the treatment of various ailments.

## Figures and Tables

**Table 1:** Effect of *Adansonia digitales* leaves extract on body weight (gm) of diabetic rats.

Treatment		Time in week			
		0	2	4	6
Normal control		183.00±3.11	234.13±7.47	269.56±11.43	280.00±7.00

Diabetic control		193.42±8..63	190.00±5.48^N^	181.00±8.21^N^	172.00±9.00^N^

Diabetic + GBC (5mg/Kg)		177.00±6.10	230.66±4.92*∗*	254.00±7.00*∗*	266.03±8.43

*Diabetic* + Leaf extract	200mg/Kg	200.23±5.79	210.00.±3.79	230.14±8.00 *∗*	236.87±10.00^N^
	400mg/Kg	190.76±7.14	240.89±6.84	255.76±11.05*∗*	250.88±8.69

All the data has been expressed as mean ± SEM of eight independent samples from each group (*n* = 8) analyzed by SPSS 13.0 version. The treated groups are CN (control normal), DM (diabetic or group II). DM+E200: diabetic rats treated with 200mg/Kg of leaf extract (group III); DM+E400: diabetic rats treated with 400mg/Kg of leaf extract; DM+GBC (group IV): diabetic rats treated with glibenclamide (group V).

N: significant difference from normal control.

*∗*: significant difference from positive control (untreated diabetic).

@: significant difference from the reference drug, glibenclamide.

**Table 2:** Effect of *Adansonia digitales* leaves extract on blood cholesterol level (mg/dl) of diabetic rats.

Treatment		Time in week			
		0	2	4	6
Normal control		70.32±1.75	74.65±4.27	82.56±4.93	75.69±4.83

Diabetic control		161.86±3.99^N^	168.40±8.11^N^	177.61±5.65^N^	180.36 ±7.44^N^

Diabetic +GBC (5 mg/Kg)		170.38±5.00^N^	140.17±7.66^N^*∗*	115.45±3.22^N^*∗*	77.15±3.85*∗*

*Diabetic* + Leaf extract	200 mg/Kg	176.91±9.22^N^	169.20±5.88^N^ @	154.03±4.64^N^*∗*@	142.19±5.23^N^*∗*@
	400 mg/Kg	189.00 ± 8.02^N^	150.22±3.28^N^	133.45±6.57^N^*∗*	91.78±5.61*∗*

All the data has been expressed as mean ± SEM of eight independent samples from each group (*n* = 8) analyzed by SPSS 13.0 version. The treated groups are CN (control normal), DM (diabetic or group II). DM+E200: diabetic rats treated with 200mg/Kg of leaf extract (group III); DM+E400: diabetic rats treated with 400mg/Kg of leaf extract; DM+GBC (group IV): diabetic rats treated with glibenclamide (group V).

N: significant difference from normal control.

*∗*: significant difference from positive control (untreated diabetic).

@: significant difference from the reference drug, glibenclamide.

**Table 3:** Effect of *Adansonia digitales* leaves extract on blood triglycerides level (mg/dl) of diabetic rats.

Treatment		Time in week			
		0	2	4	6
Normal control		57.13±1.70	64.18±4.89	60.33±2.74	55.93±1.06

Diabetic control		150.12±8.0^N^	160.55±7.44^N^	167.48 ±5.9^N^	161.07±3.83^N^

Diabetic + GBC (5 mg/Kg)		144.68±6.34^N^	110.00±4.35^N^*∗*	92.46±3.71N*∗*	68.70±3.38^N^*∗*

*Diabetic* + *Leaf extract*	200 mg/Kg	167.20±7.63^N^	154.15±4.84^N^	145.47±6.56^N^*∗*@	120.32 ± 5.68^N^*∗*@
	400 mg/Kg	155.83±7.50^N^	136.22±6.25^N^*∗*	117.19±3.99^N^*∗*@	91.55 ± 6.00^N^*∗*@

All the data has been expressed as mean ± SEM of eight independent samples from each group (*n* = 8) analyzed by SPSS 13.0 version. The treated groups are CN (control normal), DM (diabetic or group II). DM+E200: diabetic rats treated with 200mg/Kg of leaf extract (group III); DM+E400: diabetic rats treated with 400mg/Kg of leaf extract; DM+GBC (group IV): diabetic rats treated with glibenclamide (group V).

N: significant difference from normal control.

*∗*: significant difference from positive control (untreated diabetic).

@: significant difference from the reference drug, glibenclamide.

**Table 4:** Effect *Adansonia digitales* leaf extract on blood HDL (mg/dl) of diabetic rats.

Treatment		Time in week			
		0	2	4	6
Normal control		35.54±1.35	40.58±2.19	36.14±1.26	38.91±2.17
Diabetic control		41.71 ±1.67	38.32 ±0.34	30.00±0.97	29.12 ±0.86^N^
Diabetic +GBC (5 mg/Kg)		37.65±1.46	38.16±2.54	41.66±2.03	42.15±3.96*∗*
*Diabetic* + *Leaf extract*	200 mg/Kg	45.22 ±2.66	44.15±2.41	36.11 ±0.89	31.34±1.50*∗*
	400 mg/Kg	40.37 ±1.67	45.61 ±1.09	37. 06 ±1.63	38.85±2.17*∗*

All the data has been expressed as mean ± SEM of eight independent samples from each group (*n* = *8*) analyzed by SPSS 13.0 version. The treated groups are CN (control normal), DM (diabetic or group II). DM+E200: diabetic rats treated with 200mg/Kg of leaf extract (group III); DM+E400: diabetic rats treated with 400mg/Kg of leaf extract; DM+GBC (group IV): diabetic rats treated with glibenclamide (group V).

N: significant difference from normal control.

*∗*: significant difference from positive control (untreated diabetic).

@: significant difference from the reference drug, glibenclamide.

**Table 5:** Effect of *Adansonia digitales* leaves extract on blood LDL (mg/dl) in diabetic rats.

Treatment		Time in week			
		0	2	4	6
Normal control		19.73±0.54	22.05±1.13	20.48±1.05	18.86±0.73

Diabetic control		110.00±1.91^N^	100.25±3.44^N^	96.14 ±2.00^N^	98.45 ±3.85^N^

Diabetic + GBC (5 mg/Kg)		97.80±5.26^N^	66.48±3.15^N^*∗*	41.96±2.69^N^*∗*	33.07±1.72^N^*∗*

Diabetic +Leaf extract	200 mg/Kg	100.88±2.05^N^	93.22 ±3.03^N^ @	80.32±2.11^N^*∗*@	61.30±4.01^N^*∗*@
	400 mg/Kg	102.48 ±3.97^N^	77.32 ±1.74^N^*∗*@	51.86±2.08^N^*∗*@	39.65. ±1.07^N^*∗*

All the data has been expressed as mean ± SEM of eight independent samples from each group (*n* = 8) analyzed by SPSS 13.0 version. The treated groups are CN (control normal), DM (diabetic or group II). DM+E200: diabetic rats treated with 200mg/Kg of leaf extract (group III); DM+E400: diabetic rats treated with 400mg/Kg of leaf extract; DM+GBC (group IV): diabetic rats treated with glibenclamide (group V).

N: significant difference from normal control.

*∗*: significant difference from positive control (untreated diabetic).

@: significant difference from the reference drug, glibenclamide.

**Table 6:** Effect of *Adansonia digitales* leaf extract on blood cell count, glycosylated hemoglobin, and erythropoietin of diabetic rats.

Parameters	Groups				
	Normal control	Diabetic control	Diabetic + Leaf extract		Diabetic + GBC 0.5 mg/Kg
			200 mg/kg	400 mg/kg	
RBCs count (x10^6^/ml)	7.50±0.46	4.00 ±0.25^N^	4.70±0.11^N^*∗*@	5.33±0.25^N^*∗*@	6.11 ±0.16^N^*∗*

WBCs count (x10^5^/ml)	5.40 ±0.23	12.50±0.65^N^	9.76±0.47^N^*∗*@	8.00±0.41^N^*∗*	7.65±0.58^N^*∗*

Hb level (g/dl)	16.00±1.04	11.85±0.49^N^	12.50±0.23^N^*∗*@	14.78±0.54^N^*∗*	14.90±0.36^N^*∗*

PCV (%)	50.00±2.96	35.19±1.28^N^	44.00±2.18^N^*∗*@	53.39±3.21*∗*	54.00±3.25*∗*

GHb (%)	4.56±0.07	13.00±0.48^N^	8.60 ±0.54^N^*∗*@	7.00±0.16^N^*∗*	7.50±0.68^N^*∗*

EPO (ng/ml)	2.78±0.04	0.58±0.01^N^	1.34±0.05^N^*∗*@	1.86±0.03^N^*∗*@	2.20±0.06^N^*∗*

All the data has been expressed as mean ± SEM of eight independent samples from each group (*n* = 8) analyzed by SPSS 13.0 version. The treated groups are CN (control normal), DM (diabetic or group II). DM+E200: diabetic rats treated with 200mg/Kg of leaf extract (group III); DM+E400: diabetic rats treated with 400mg/Kg of leaf extract; DM+GBC (group IV): diabetic rats treated with glibenclamide (group V).

N: significant difference from normal control.

*∗*: significant difference from positive control (untreated diabetic).

@: significant difference from the reference drug, glibenclamide.

GBC: glibenclamide; GHb: glycosylated hemoglobin; EPO: Erythropoietin.

## Data Availability

The data used to support the findings of this study are included within the article.

## Abbreviations

CAT: Catalase
IL-6: Interleukin 6
HDL: High-density lipoprotein
GSH: Reduced glutathione
MDA: Malondialdehyde
LDL: Low-density lipoprotein
PCV: Packed cell volume
SOD: Superoxide dismutase
STZ: Streptozotocin
TC: Total Cholesterol
TG: Triglycerides
TNF-*α*: Tumor necrosis factor-alpha.
